# Salmonella enterica Serovar Typhimurium Exploits Inflammation to Compete with the Intestinal Microbiota

**DOI:** 10.1371/journal.pbio.0050244

**Published:** 2007-08-28

**Authors:** Bärbel Stecher, Riccardo Robbiani, Alan W Walker, Astrid M Westendorf, Manja Barthel, Marcus Kremer, Samuel Chaffron, Andrew J Macpherson, Jan Buer, Julian Parkhill, Gordon Dougan, Christian von Mering, Wolf-Dietrich Hardt

**Affiliations:** 1 Institute of Microbiology, Swiss Institute of Technology Zurich, Zurich, Switzerland; 2 Wellcome Trust Sanger Institute, Hinxton, Cambridge, United Kingdom; 3 Department of Mucosal Immunity, Helmholtz Centre for Infection Research, Braunschweig, Germany; 4 Technical University Munich, Munich, Germany; 5 Bioinformatics Group, Institute of Molecular Biology, University of Zurich, Zurich, Switzerland; 6 Faculty of Health Sciences, McMaster University, Hamilton, Ontario, Canada; Harvard University, United States of America

## Abstract

Most mucosal surfaces of the mammalian body are colonized by microbial communities (“microbiota”). A high density of commensal microbiota inhabits the intestine and shields from infection (“colonization resistance”). The virulence strategies allowing enteropathogenic bacteria to successfully compete with the microbiota and overcome colonization resistance are poorly understood. Here, we investigated manipulation of the intestinal microbiota by the enteropathogenic bacterium Salmonella enterica subspecies 1 serovar Typhimurium (*S.* Tm) in a mouse colitis model: we found that inflammatory host responses induced by *S.* Tm changed microbiota composition and suppressed its growth. In contrast to wild-type *S.* Tm, an avirulent *invGsseD* mutant failing to trigger colitis was outcompeted by the microbiota. This competitive defect was reverted if inflammation was provided concomitantly by mixed infection with wild-type *S.* Tm or in mice (IL10^−/−^, VILLIN-HA^CL4-CD8^) with inflammatory bowel disease. Thus, inflammation is necessary and sufficient for overcoming colonization resistance. This reveals a new concept in infectious disease: in contrast to current thinking, inflammation is not always detrimental for the pathogen. Triggering the host's immune defence can shift the balance between the protective microbiota and the pathogen in favour of the pathogen.

## Author Summary

A dense microbial community colonizes the intestinal tract of mammals, contributing to health and nutrition and conferring efficient protection against most pathogenic intruders. Intestinal pathogens can overcome this colonization resistance and cause disease; however, the mechanisms used to do this are still elusive. In this study we analyzed intestinal infection by the model pathogen Salmonella enterica subspecies 1 serovar Typhimurium (*S.* Tm). We show that the virulent wild-type pathogen overcomes colonization resistance by inducing the host's inflammatory immune response and exploiting it for its purpose. In contrast, an avirulent *Salmonella* mutant defective in triggering inflammation was unable to overcome colonization resistance by itself. Colonization by this mutant was restored if inflammation was provided concomitantly, in mice with inflammatory bowel disease (genetic and inducible) or by co-infection with wild-type *S*. Tm. These findings reveal a previously unrecognized strategy by which pathogenic bacteria overcome colonization resistance: abusing the host's inflammatory immune response to gain an edge against the normal microbial community of the gut. This represents a first step towards unravelling the molecular mechanisms underlying this three-way interaction of host, microbiota, and pathogens.

## Abstract 3

Inducing inflammation is key to the ability of the virulent pathogen *Salmonella enterica* serovar Typhimurium to outcompete the protective resident microbiota in a race to colonize the gut.

## Introduction

**Figure 1:**
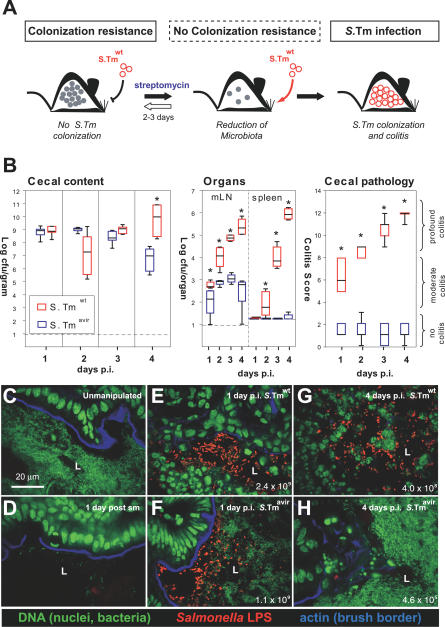
Microbiota Outcompete *S.* Tm^avir^ but not *S.* Tm^wt^ (A) Streptomycin-treated mouse model. The antibiotic transiently reduces the microbiota (grey circles) in the lumen of the large intestine, reduces colonization resistance, and allows colonization and induction of colitis by *S.* Tm^wt^. (B) Streptomycin-treated C57BL/6 mice (*n =* 7 per group) were infected with *S.* Tm^avir^ (blue) or *S.* Tm^wt^ (red; 5 × 10^7^ CFU i.g.). At indicated time points mice were sacrificed, *S.* Tm loads were determined in cecal content, mLN, and spleen, and cecal pathology was scored. Detection limits (dotted lines): cecal content, 10 CFU/g; mLN, 10 CFU/organ; spleen, 20 CFU/organ. *, *p* ≤ 0.05; statistically significant difference between *S.* Tm^avir^ and *S.* Tm^wt^. Boxes indicate 25th and 75th percentiles, black bars indicate medians, and whiskers indicate data ranges. (C–H) Representative confocal fluorescence microscopy images of cecum tissue sections from the mice shown in (B). Nuclei and bacterial DNA are stained by Sytox green (green), the epithelial brush border actin by Alexa-647-phalloidin (blue), and extracellular *S.* Tm in the intestinal lumen by anti–*S*. Tm LPS antiserum (red). Normal microbiota in unmanipulated mice (C), microbiota 1 d after streptomycin (sm) treatment (D), streptomycin-treated mice infected for 1 or 4 d with *S.* Tm^avir^ or *S.* Tm^wt^ (E–H). The *S*. Tm colonization levels are indicated (CFU/g); L, cecum lumen.

The evolution of pathogenic microorganisms has been shaped to a great extent by their interaction with cognate host species. Colonization is the first step of any infection. For enteropathogenic bacteria, this poses a formidable task as the target host organ is already colonized by a dense microbial community, the microflora, or “microbiota”. Intestinal colonization by microbiota begins immediately after birth and lasts for life. In a healthy intestine, the microbiota is quite stable, and its gross composition at higher taxonomic levels is similar between individuals, and even between humans and mice [1]. The intestinal ecosystem is shaped by symbiotic interactions between the host and the microbiota. Microbiota composition is influenced by nutrient availability, local pH, and possibly also by the host's immune system [2]. Conversely, the microbiota optimizes nutrient utilization [3,4], and boosts maturation of intestinal tissues and the intestinal immune system [5–7]. In addition, the microbiota provides an efficient barrier against infections (“colonization resistance”), which must be overcome by enteropathogenic bacteria. It is poorly understood how enteropathogens can achieve that task. Here, we used Salmonella enterica subspecies 1 serovar Typhimurium (*S.* Tm) and a mouse colitis model to study strategies by which enteropathogenic bacteria break colonization resistance. *S.* Tm infects a broad range of animal species and is a frequent cause of intestinal infections in the human population. The normal murine microbiota provides colonization resistance and prevents intestinal colonization upon oral *S*. Tm infection. Oral treatment with the antibiotic streptomycin (20 mg of streptomycin intragastric [i.g.]) transiently reduces the microbiota by >80% and disrupts colonization resistance for a period of 24 h [8,9]. The residual microbiota re-grows within 2–3 d, and colonization resistance is re-established ([9]; unpublished data). These studies have provided the basis for a “streptomycin mouse model” for *Salmonella* enterocolitis [10]: 1 d after streptomycin treatment, oral infection with *S*. Tm leads to efficient colonization of the murine intestine, especially the cecum and the colon (approximately 10^9^ colony-forming units [CFU]/gram; Figures 1A and S1) [8,9,11]. Wild-type *S*. Tm (*S.* Tm^wt^) triggers pronounced intestinal inflammation (colitis) and colonizes the intestinal lumen at high densities over extended periods of time [8,10–12]. This “streptomycin mouse model” can be used to study bacterial virulence factors required for colonization and triggering of intestinal inflammation. For example, *S.* Tm strains lacking the two virulence-associated type III secretion systems (e.g., *S.* Tm Δ*invG sseD::aphT* [*S.* Tm^avir^] [13]) cannot trigger colitis. In addition, these mutants were found to colonize the murine intestine only transiently [11,13]. The reason for this colonization defect has remained elusive.

To explore this, we analyzed microbiota compostition in *S.* Tm^wt^– and *S.* Tm^avir^–infected mice and the role of inflammation for *Salmonella* colonization and competition against the intrinsic microbiota. We found that inflammation shifts the balance between the protective microbiota and the pathogen *S.* Tm in favour of the pathogen. This principle might apply to various other pathogens and therefore constitute a novel paradigm in infectious biology.

## Results

### *S.* Tm^avir^ but Not *S.* Tm^wt^ Is Outcompeted by Commensal Microbiota

First, we confirmed the differential colonization efficiency of *S*. Tm^wt^ and *S.* Tm^avir^ in the streptomycin mouse model. Unlike *S*. Tm^wt^, intestinal *S*. Tm^avir^ colonization levels decreased significantly by day 4 post-infection (p.i.) in a highly reproducible fashion (Figure 1B). This coincided with re-growth of the microbiota as revealed by immunofluorescence microscopy (Figure 1C–1H). By anaerobic culture, DNA isolation, and 16S rRNA gene sequencing, high densities of characteristic members of the intestinal microbiota (Clostridium spp., Bacteroides spp., and Lactobacillus spp. [14]) were found in *S.* Tm^avir^–infected, but not in *S.* Tm^wt^–infected, animals at day 4 p.i. (Table 1). Both the *S*. Tm/microbiota ratio and the composition of the microbiota itself differed between mice infected with *S.* Tm^avir^ and *S.* Tm^wt^. These data demonstrated that residual microbiota surviving the streptomycin treatment can re-grow, outcompete *S.* Tm^avir^, and thereby re-establish colonization resistance. In contrast, *S.* Tm^wt^ can suppress re-growth of the residual microbiota. Therefore, the streptomycin mouse model allows study of the principal mechanisms by which enteropathogens manipulate the intestinal ecosystem.

**Table 1:**
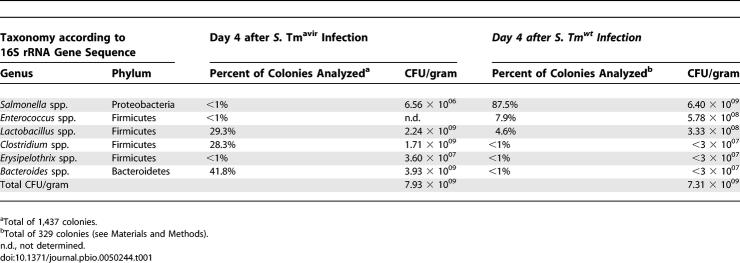
Bacterial Genera Recovered by Anaerobic Culture from *S.* Tm Infected Mice

### *S.* Tm^wt^ Alters Composition of the Microbiota in the Streptomycin Mouse Model

**Figure 2:**
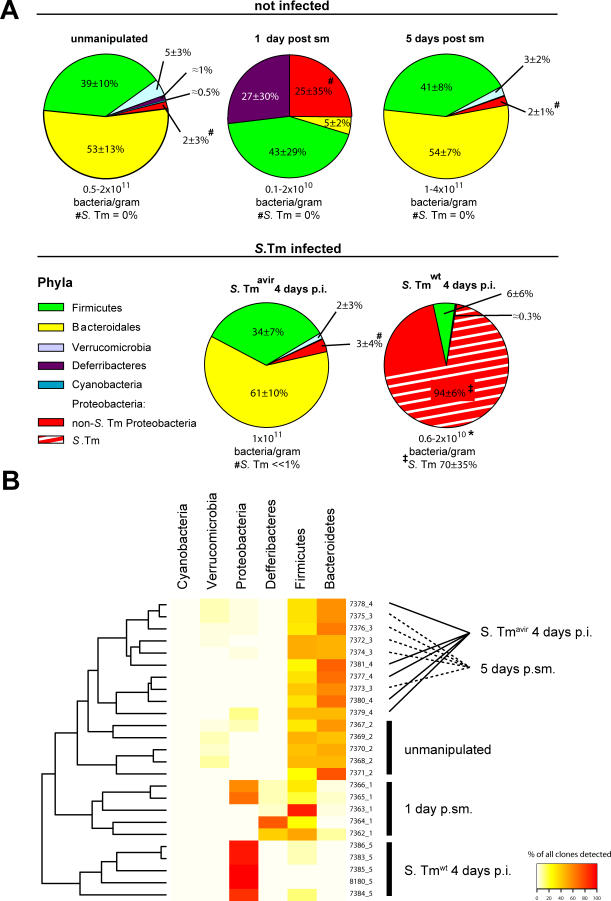
16S rRNA Gene Sequence Analysis of Microbiota Manipulation by *S.* Tm^wt^ and *S.* Tm^avir^ in the Streptomycin Mouse Model Cecal contents were recovered from unmanipulated mice, mice at days 1 or 5 after streptomycin treatment (20 mg i.g.), and streptomycin-treated mice 4 d after infection with *S.* Tm^avir^ and *S.* Tm^wt^ (5 × 10^7^ CFU i.g.; all *n =* 5). Total DNA was extracted, and bacterial 16S rRNA genes were PCR-amplified using universal bacterial primers, cloned, and sequenced (approximately 100 sequences per animal; five animals per group; see Materials and Methods). (A) Pie diagrams showing the microbiota composition at the phylum level. Numbers below the diagrams indicate bacteria/gram cecal content as defined by Sytox green staining. *The lower bacterial density in *S.* Tm^wt^–infected mice is attributable to a high proportion of cellular debris in the intestinal lumen (see Figure 1G). ^#^In these groups no *Salmonella* 16S rRNA genes were identified. ^‡^Proteobacterial sequences belonged to *Salmonella (E. coli)* in the following percentages: 91 (1), 15 (70), 87 (11), 55 (38), and 100 (0). See also Table S1. (B) Visual depiction of the microbiota composition of individual mice. The animals were grouped based on the similarity of their microbiota composition at the phylum level (using the Canberra distance as metric). The resulting groupings are depicted as a dendrogram, and observed phylum counts for each mouse are shown as a heat map (0%–100% of all identified 16S rRNA gene sequences). Labels indicate unique mouse identifier numbers. The experimental groups are indicated. p.sm., post–streptomycin treatment.

To better characterize the effect of *S*. Tm on microbiota composition, we employed 16S rRNA gene sequencing (see Materials and Methods). This method allows a quantitative comparison of microbial communities, including bacterial species that cannot be cultivated in vitro. The analysis comprised five different groups of mice and addressed the effect of the streptomycin pretreatment per se as well as the effect of *S.* Tm^avir^ and *S.* Tm^wt^ infection on microbiota composition (Figure 2).

In line with published data, a large fraction of the murine microbiota in unmanipulated mice belonged to either the Firmicutes (including Clostridium spp. and Lactobacillus spp.; 39% ± 10%) or the Bacteroidales (53% ± 13%; Figure 2) [1,15–17]. Streptomycin treatment reduced the global density of the microbiota by approximately 90% (Figure 2; see also Figure 1C and 1D) and changed its relative composition (Figure 2A and 2B; Table 2). The composition of the remaining microbiota varied substantially between individual members of this group (Figure 2B). Most likely, this is attributable to the unstable situation created by the antibiotic and may arise from slight animal-to-animal variations in the timing or speed of the gut passage of the antibiotic and/or from species-specific differences in antibiotic susceptibility and rate of re-growth.

**Table 2:**
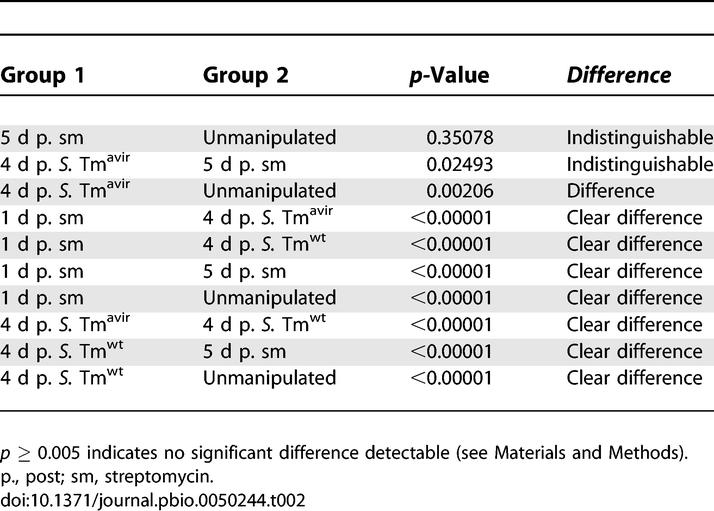
Phylum-Level Comparison of Microbiota in Streptomycin-Treated *S.* Tm–Infected Mice from Experiment Described in Figure 2

Five days after the antibiotic treatment, the microbiota had re-grown to normal density and microbiota composition, at least at the phylum level (Figure 2A and 2B; Table 2; *p* = 0.35078). Infection with *S*. Tm^avir^ did not interfere detectably with re-growth of the normal microbiota in the streptomycin-pretreated mouse model (Figure 2B; Table 2).

In contrast, *S*. Tm^wt^ significantly altered the cecal microbiota composition (Figure 2A and 2B; Table 2; *p* < 0.00001). Proteobacterial 16S rRNA gene sequences represented >90% of all sequences, and Salmonella spp. generally represented the most prominent (up to 100%) proteobacterial species in the *S*. Tm^wt^–infected animals. These observations were confirmed by fluorescence in situ hybridization (FISH) of fixed cecal content (Figure S2). This demonstrates that *S.* Tm^wt^ interferes with microbiota re-growth and represents the predominant species at day 4 p.i.

It should be noted that other proteobacterial species (e.g., Escherichia coli) were also present in significant numbers in the cecum of most *S*. Tm^wt^–infected animals (Figure 2A). These proteobacterial strains are low abundance members of the normal gut microbiota of our mouse colony (<10^7^ CFU/g of cecal content). In many mice the proportion of these commensal proteobacterial species increased concomitant with the *S.* Tm^wt^ infection. This suggests that other bacterial species closely related to *S*. Tm may also be able to benefit from the *S*. Tm^wt^–triggered inflammation. Further work will be required to address this issue.

**Figure 3:**
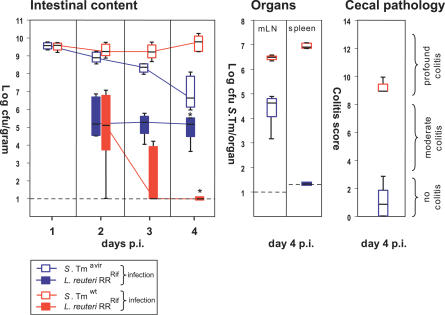
*S.* Tm^wt^ Can Suppress Colonization with L. reuteri RR^Rif^ Groups of streptomycin-treated mice (*n =* 5) were first infected with *S.* Tm^avir^ or *S.* Tm^wt^ (5 × 10^7^ CFU i.g.) and inoculated 1 d later with L. reuteri RR^Rif^ (8 × 10^6^ CFU i.g.). Colonization levels were monitored in the feces (2 and 3 d p.i.), the cecal content (4 d p.i.), the mLN, and the spleen. Box plots show *S.* Tm^avir^ (open blue boxes), *S.* Tm^wt^ (open red boxes), L. reuteri RR^Rif^ in *S.* Tm^avir^–infected mice (filled blue boxes), and L. reuteri RR^Rif^ in *S.* Tm^wt^–infected mice (filled red boxes). In all groups cecal pathology was scored at day 4 p.i. *, p ≤ 0.05; statistically significant difference in L. reuteri RR^Rif^ colonization between *S.* Tm^avir^– and *S.* Tm^wt^–infected mice. *L. reuteri* RR^Rif^ was not detected in mLN and spleen. Boxes indicate 25th and 75th percentiles, black bars indicate medians, and whiskers indicate data ranges.

The observed changes in microbiota growth in *S.* Tm^wt^–infected mice were verified in a competitive infection experiment with a specific member of the microbiota. For this purpose we selected a rifampicin-resistant variant of Lactobacillus reuteri strain RR (L. reuteri RR^Rif^). This strain was isolated as a commensal from our mouse colony. Streptomycin-treated mice were infected i.g. with either *S.* Tm^wt^ or *S.* Tm^avir^ (5 × 10^7^ CFU i.g.) and gavaged 1 d p.i. with L. reuteri RR^Rif^ (8 × 10^6^ CFU i.g.). L. reuteri RR^Rif^ colonized the *S.* Tm^avir^–infected mice at levels of 10^5^–10^6^ CFU/g of intestinal content. In *S.* Tm^wt^–infected mice, similar L. reuteri RR^Rif^ colonization levels were observed at day 2 p.i., but colonization levels declined below the detection limit by day 4 p.i. (*p =* 0.008; Figure 3). Thus, alteration of microbiota composition by *S.* Tm^wt^ can be demonstrated at the level of a single bacterial strain.

### Intestinal Inflammation Is Sufficient to Enhance Colonization by *S.* Tm^avir^

**Figure 4:**
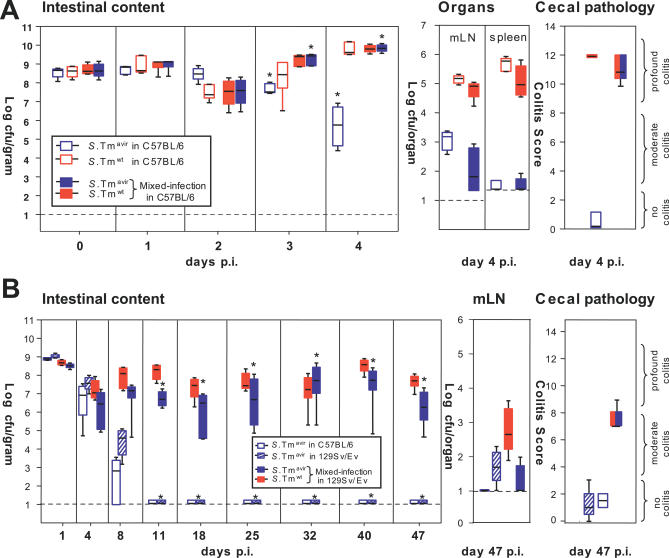
*S.* Tm^wt^–Induced Inflammation Enhances Colonization of *S.* Tm^avir^ (A) Mixed infection with *S.* Tm^wt^ complements the colonization defect of *S.* Tm^avir^. Streptomycin-treated C57BL/6 mice (*n =* 5/group) were infected with 5 × 10^7^ CFU i.g. of *S.* Tm^avir^ only (open blue boxes), *S.* Tm^wt^ only (open red boxes), or a 1:1 mixture of the two strains (filled blue and red boxes, respectively). Colonization was measured in the feces (days 0–3 p.i.) and the cecal content (day 4 p.i.) (left panel). Colonization of mLN and spleen (middle panel) and cecal pathology (right panel) were determined at day 4 p.i. (B) Mixed infection with *S.* Tm^wt^ complements the colonization defect of *S.* Tm^avir^ in a chronic *Salmonella* colitis model (129Sv/Ev mice). Groups of streptomycin-treated mice (NRAMP^+^ 129Sv/Ev mice raised by C57BL/6 foster mice; *n =* 4 per group) were infected with 5 × 10^7^ CFU i.g. of *S.* Tm^avir^ only (blue-striped boxes) or a 1:1 mixture of *S.* Tm^avir^ and *S.* Tm^wt^ (filled blue and red boxes, respectively). One additional control group (four streptomycin-treated C57BL/6 mice) was infected with *S.* Tm^avir^ (5 × 10^7^ CFU i.g.; open blue boxes). Colonization was measured in the feces (days 1–40 p.i.) and the cecal content (day 47 p.i.) (left panel). Colonization of mLN and spleen (middle panel) and cecal pathology (right panel) were analyzed at day 47 p.i. Boxes indicate 25th and 75th percentiles, black bars indicate medians, and whiskers indicate data ranges.

The above findings prompted us to investigate whether there is a cause-and-effect relationship between triggering of inflammation and enhanced colonization by *S.* Tm. In this case one would predict that *S.* Tm^avir^ (which cannot trigger inflammation) competes successfully with the microbiota if inflammation is triggered by other means. Three different experimental approaches lent evidence for this hypothesis:

First, we analyzed whether inflammation induced by *S.* Tm^wt^ improved *S.* Tm^avir^ colonization efficiency. Earlier experiments had shown that infections with 1:1 mixtures of *S.* Tm^wt^ and attenuated mutants led to full-blown colitis (Figure 4A and data not shown). Thus, streptomycin-treated mice were infected with a 1:1 mixture of *S.* Tm^wt^ and *S.* Tm^avir^ (a total of 5 × 10^7^ CFU i.g.). Control groups were infected with *S.* Tm^wt^ or *S.* Tm^avir^ only (5 × 10^7^ CFU i.g.; Figure 4A). Pronounced colitis was observed in all animals infected with *S.* Tm^wt^ and the *S.* Tm^wt^–*S.* Tm^avir^ mixture, but not in animals infected with *S.* Tm^avir^ alone. Furthermore, *S.* Tm^avir^ was severely defective at colonizing lymph nodes and spleen in single and mixed infections. Despite its non-pathogenic phenotype, *S.* Tm^avir^ colonized the cecal lumen up to wild-type levels in mixed infections with *S.* Tm^wt^. Thus, concomitant colitis created favourable conditions in the intestinal lumen that suppressed microbiota regrowth and rescued *S.* Tm^avir^ colonization in tandem. This was confirmed in long-term infection experiments using 129Sv/Ev mice, which develop a chronic form of colitis (Figures 4B and S3) [12].

**Figure 5:**
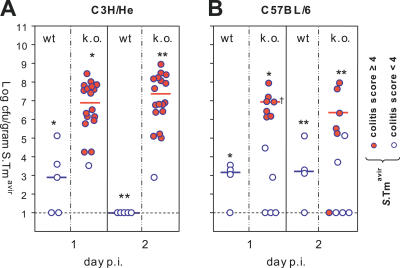
Intestinal Inflammation in IL10^−/−^ Mice Enhances Colonization of *S.* Tm^avir^ (A) C3H/HeJBir^IL10−/−^ (*n =* 18) and C3H/HeJ control animals (*n =* 5) were infected with *S.* Tm^avir^ (5 × 10^7^ CFU i.g.; no streptomycin treatment). *S.* Tm^avir^ colonization was analyzed in feces (day 1 p.i.) and cecum content (day 2 p.i.), and cecal pathology was scored (see Material and Methods). Open blue circles indicate mice with colitis score < 4; blue circles with red filling indicate mice with colitis score ≥ 4. *, *p =* 0.03; **, *p =* 0.004. (B) C57BL/6^IL10−/−^ (*n =* 12) and C57BL/6 control animals (*n =* 4) were infected with *S.* Tm^avir^ (5 × 10^7^ CFU i.g.; no streptomycin treatment). *S.* Tm^avir^ colonization and cecal pathology were analyzed as described above. Open blue circles indicate mice with colitis score < 4; blue circles with red filling indicate mice with colitis score ≥ 4. *, *p =* 0.006; **, *p =* 0.016. †One animal was sacrificed at the end of day 1 p.i. for humane reasons.

Next, we studied whether cecal inflammation per se (in absence of *S.* Tm^wt^) could enhance *S.* Tm^avir^ colonization. For this purpose we employed knockout mouse models lacking the key anti-inflammatory cytokine IL10. Depending on the exact genetic background and the composition of the microbiota, these animals develop colitis spontaneously earlier (week 6; C3H/HeJBir^IL10−/−^ model [18]) or later in life (week 30–50; C57BL/6^IL10−/−^ model [19]). To test the effect of pre-existing colitis on *S.* Tm^avir^ colonization, groups of 8-wk-old C3H/HeJBir^IL10−/−^ mice and C3H/He control mice were infected (5 × 10^7^ CFU of *S.* Tm^avir^ i.g.; no streptomycin treatment). Fecal shedding (day 1 p.i.), colonization, and colitis (day 2 p.i.) were analyzed. Colonization of the intestinal lumen by *S.* Tm^avir^ was significantly enhanced in mice displaying colitis (day 2 p.i., *p =* 0.016; Figures 5A, S3, and S4). Similar observations were made using the C57BL/6^IL10−/−^ model. In C57BL/6^IL10−/−^ mice, the onset of colitis is quite random and varies anywhere from 30 to 50 wk even between littermates. Accordingly, we infected C57BL/6^IL10−/−^ littermates 30–50 wk of age (5 × 10^7^ CFU of *S.* Tm^avir^ i.g.; no streptomycin treatment). Again, colonization of the intestinal lumen by *S.* Tm^avir^ was enhanced in littermates displaying colitis (day 1 p.i., *p =* 0.016; Figures 5B, S3, and S4). This suggested that inflammation per se can enhance *S.* Tm^avir^ colonization.

**Figure 6:**
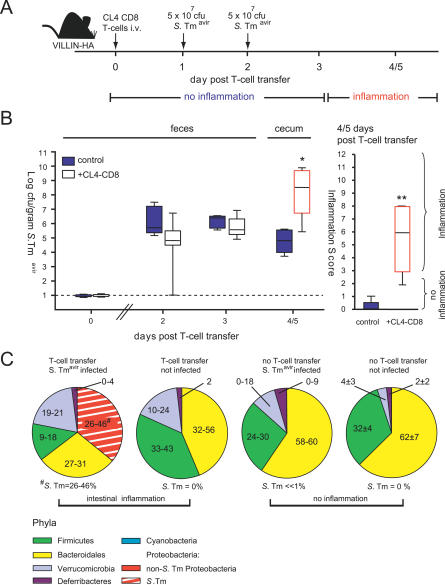
Gut Inflammation in the VILLIN-HA^CL4-CD8^ Model Boosts *S.* Tm^avir^ Colonization (A) The VILLIN-HA^CL4-CD8^ model including the time course of intestinal inflammation and the infection regime employed in the experiment shown below. (B) Gut colonization by *S.* Tm^avir^ is enhanced when inflammation occurs. Seven VILLIN-HA mice received 4 × 10^6^ CL4-CD8 T cells (open white boxes) at day 0. Five unmanipulated VILLIN-HA transgenic mice served as control (blue boxes; no T cell transfer). Both groups of mice were inoculated with 5 × 10^7^ CFU i.g. of *S.* Tm^avir^ at days 1 and 2. *S.* Tm^avir^ colonization was measured in the feces (days 1 and 2 p.i.). When symptoms of colitis (weight loss and diarrhoea) were observable in the animals from the experimental group (day 4/5), mice were sacrificed and *S.* Tm^avir^ loads in the cecal content (left) as well as cecal pathology (right) were determined (open red boxes indicate inflammation). *, *p =* 0.01; **, *p =* 0.003. Boxes indicate 25th and 75th percentiles, black bars indicate medians, and whiskers indicate data ranges. (C) Pie diagrams showing the fecal microbiota composition at the phylum level. The average for *n* = 2 animals per group (approximately 100 16S rRNA gene sequences per animal) is shown for all groups except “no T cell transfer, not infected”, for which the average for four mice is shown. Information at higher taxonomic resolution is provided in Table S1. The *p*-values are shown in Table S2.

To verify this hypothesis we employed the alternative, recently developed VILLIN-HA^CL4-CD8^ mouse model for T cell–induced colitis [20]. This model employs VILLIN-HA transgenic mice expressing the HA epitope in the gut epithelium and T cells (CD8^+^; HA-directed α/β T cell receptor; from CL4-TCR transgenic mice) recognizing the HA epitope. Adoptive transfer of these T cells into VILLIN-HA transgenic mice results in severe inflammation of the small and the large intestine at 4–5 d post-transfer (Figure 6A) [20]. This model was of particular interest because intestinal inflammation develops quickly, occurs in the majority of animals, and does not involve i.g. treatment with chemicals that might themselves influence the microbiota–pathogen competition.

To study the impact of inflammation on *S.* Tm^avir^ colonization we infected VILLIN-HA transgenic mice receiving CL4-CD8 T cells and unmanipulated VILLIN-HA control mice. In the unmanipulated VILLIN-HA mice (no T cells transferred), no intestinal inflammation was apparent and *S.* Tm^avir^ colonization efficiency was low (Figure 6B). In contrast, the animals receiving 4 × 10^6^ CL4-CD8 T cells (VILLIN-HA^CL4-CD8^ mice) developed intestinal inflammation 4 or 5 d after T cell transfer, and *S.* Tm^avir^ efficiently colonized the intestine of these animals (*p =* 0.01; Figure 6B). It should be noted that the initial colonization by *S.* Tm^avir^ was poor (fecal samples at days 2 and 3 after T cell transfer) and that the onset of efficient *S.* Tm^avir^ colonization closely correlated with the onset of the intestinal inflammation (day 4–5 after T cell transfer [20]). At this stage, *“Salmonella”* sequences represented 26%–46% of all bacterial 16S rRNA genes recovered from the cecal contents (Figure 6C). This confirmed that colitis per se creates conditions in the gut skewing the competition between Salmonella spp. and the microbiota in favour of the pathogen.

As additional controls, we analyzed the fecal microbiota composition of unmanipulated VILLIN-HA transgenic mice (*n* = 4) and non-infected VILLIN-HA transgenic mice (*n* = 2) at day 4 after CL4-CD8 T cell transfer (Figures 6C and S6; Table S2). The latter two animals showed intestinal inflammation comparable to that in mice that received CL4-CD8 T cells and *S.* Tm^avir^ (data not shown). At the phylum level, we did not detect any significant differences between the microbiota recovered from the feces of the unmanipulated mice (no gut inflammation), the VILLIN-HA transgenic mice that had received CL4-CD8 T cells (gut inflammation), and the *S.* Tm^avir^–infected VILLIN-HA transgenic mice that had not received CL4-CD8 T cells (no gut inflammation). These data suggest that inflammation per se does not drastically alter the gross gut flora composition (at least not in the short term). Further work is required to determine whether the loss of colonization resistance in the inflamed VILLIN-HA transgenic mice is attributable to suppression of some particular, low abundance member(s) of the microbiota.

Finally, our data show that *S.* Tm^avir^ colonization efficiency in the murine intestine is restricted by the intestinal microbiota. In the absence of microbiota, *S*. Tm^avir^ should colonize efficiently. This was confirmed in germ-free mice that lack microbiota in the first place. *S.* Tm^avir^ colonized the large intestine of germ-free mice at wild-type levels up to day 4 p.i. (approximately 10^9^ CFU/g) but did not cause colitis (Figure S5). Thus, *S.* Tm^avir^ efficiently colonizes the murine intestine as long as competing microbiota is lacking. Furthermore, inflammation is not required for colonizing the intestinal lumen in the absence of microbiota. However, it should be noted that germ-free mice represent a useful but highly artificial tool. In natural habitats, Salmonella spp. always encounters a dense intestinal microbiota, and intestinal colonization will be enhanced by the triggering of inflammation.

## Discussion

**Figure 7:**
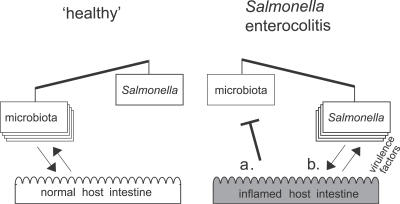
Working Model for the Microbiota–Host–Pathogen Interaction in Health and Disease Colonization resistance (or lack thereof) results from growth competition between microbiota and incoming pathogens. Host responses can skew growth conditions in the intestinal lumen in either direction. Left: the normal microbiota is shaped by mutually beneficial interactions with the intestinal mucosa and mediates colonization resistance against incoming pathogens. Right: *S*. Tm employs specific virulence factors for triggering colitis. Inflammation alters the luminal conditions and shifts the growth competition in favour of the pathogen, thus alleviating colonization resistance. Inhibitory effects on the microbiota (a) and/or improved growth conditions for the pathogen (b) may be involved. Furthermore, the microbiota–pathogen growth competition can be affected by antibiotic treatment or by pre-existing intestinal inflammation.

Based on these data we propose a three-way microbiota–pathogen–host interaction model for murine *Salmonella* colitis (Figure 7). The resident microbiota and the incoming pathogen compete for growth. In a “healthy” intestine the normal microflora is shaped and stabilized by mutually beneficial interactions with the intestinal mucosa. It effectively excludes *S*. Tm^wt^ and *S*. Tm^avir^ from the intestinal lumen. Colonization resistance can be transiently alleviated by streptomycin treatment. Inflammatory host responses—triggered by specific *S*. Tm virulence factors (TTSS-1 and TTSS-2), by genetic pre-disposition (IL10^−/−^), or by T cell–inflicted damage (VILLIN-HA^CL4-CD8^ model)—alter conditions in the intestinal lumen and shift the competition in favour of the incoming pathogen. Suppression of the microbiota or enhanced pathogen growth may be involved (Figure 7). In either case, *S*. Tm^wt^ can enhance intestinal colonization via an indirect mechanism—by triggering the host's immune defence. Thus, *S*. Tm^wt^ infection involves two different steps: triggering inflammation, and surviving in and profiting from the altered ecological niche. The avirulent mutant *S*. Tm^avir^ is unable to trigger colitis but it is still capable of taking advantage of the ecological niche opened by inflammation and thus successfully competes with the microbiota if inflammation is induced by other means.

How does intestinal inflammation subvert colonization resistance? The inflammation involves increased secretion of antibacterial peptides and lectins [21,22] and mucins (B. Stecher and W. Hardt, unpublished data), phagocyte infiltration/transmigration, and release of oxygen and nitrogen radicals. Potentially, there are a number of different ways this may subvert colonization resistance. (1) Released antibacterial factors may kill or retard growth of specific members of the microbiota that would normally inhibit *S.* Tm growth in the healthy intestine. (2) There may be “commensal network disruption”, i.e., loss of one or more specific microbiota species that might be required for efficient growth of the microbiota species that slow pathogen growth in the normal, healthy intestine. These protecting species and their integration into microbiota growth networks have not been identified. (3) There may be differential defence susceptibility. Microbiota species conferring colonization resistance might be susceptible to antibacterial defences that *S.* Tm can resist. This would be in line with the discovery of numerous *S.* Tm genes that function to enhance antimicrobial peptide resistance and radical detoxification [23–25]. (4) There may be enhanced pathogen growth. The altered nutrient mix available in the inflamed gut might foster efficient pathogen replication. Under these conditions, microbiota may simply grow slower and are thus overgrown by the pathogen. The model is summarized in Figure 7. Future work will have to address which of these mechanisms contribute to subversion of gut inflammation by *S*. Tm.

Inflammation induced by *S.* Tm, self-reactive T cells, or IL-10 deficiency enhances colonization by the pathogen and reduces growth of the commensal microbiota. Other proteobacteria closely related to *S*. Tm may also benefit from inflammation (e.g., *E. coli;* see Figure 2). Thus, this principle may also apply to other enteric infections. For example, in calves, which are naturally susceptible to *Salmonella* enterocolitis, defects of *Salmonella* TTSS-2 mutants in triggering inflammation are associated with attenuation of intestinal colonization [26,27]. Similar observations were made with *Shigella flexneri, Vibrio cholerae,* and *Citrobacter rodentium,* the causative agents of bacillary dysentery, cholera, and transmissible murine colonic hyperplasia: ablation of colitis by disrupting the hosts' innate immune response or specific bacterial virulence factors coincided with reduced intestinal colonization [28–31]. Thus, intestinal inflammation and efficient colonization may be linked in a broad range of enteropathogenic infections.

Some data are available for human *Salmonella* enterocolitis. In line with findings in the murine system, antibiotics are known to reduce human colonization resistance, and altered microbiota composition is commonly observed in patients with inflammatory bowel disease (IBD) [32–34]. Furthermore, some studies suggest an increased incidence of *Salmonella* colonization in IBD patients [35–40].

Microbiota composition in IBD patients significantly differs from that in healthy controls. Currently, an imbalance in normal gut microbiota is regarded as one possible factor triggering the inflammation in Crohn disease and ulcerative colitis [41–43]. Our data suggest that the altered gut flora might not be the cause, but rather one of the many symptoms, of intestinal inflammation in IBD patients. Further investigation into this idea will be of importance for basic research exploring the aetiology and pathogenesis of Crohn disease and ulcerative colitis.

The outcome of any infection is determined through competition between the bacterial virulence factors (enhancing pathogen replication/persistence) and the host's immune defences (eliminating the pathogen). In the case of enteropathogens, which target a niche colonized by the microbiota, the virulence factors can serve an additional function that has remained unrecognized: they allow triggering of intestinal inflammation that subverts the host's immune defences for undermining colonization resistance. This may represent a common virulence strategy of enteropathogenic bacteria including *Clostridium difficile,* which is a frequent cause of antibiotic-associated colitis. In fact, inflammation may promote pathogen competitiveness at any colonized site of the human body, and pathogens infecting the respiratory tract, the uro-genital system, and the skin might also use this strategy. Molecular analysis of the complex three-way pathogen–host–microbiota interactions poses a great technological challenge for future research and promises to reveal novel avenues for determining prevention strategies and cures for infectious disease.

## Materials and Methods

### Animals.

All aspects of animal procedures were approved by local authorities and performed according to the legal requirements. Sex- and age-matched specified pathogen free (SPF) C57Bl/6 (Elévage Janvier; http://www.janvier-breedingcenter.com/), 129Sv/Ev, C3H/He (Charles River Laboratories, http://www.criver.com/), C57BL/6^IL10−/−^ [19], and C3H/HeJBir^IL10−/−^ [18] mice were held under barrier conditions at the Rodent Centre, Swiss Institute of Technology Zurich, Zurich, Switzerland, and the Biologisches Zentrallabor, University of Zurich, Zurich, Switzerland. VILLIN-HA [44] and CL4-TCR [45] transgenic mice were raised under SPF barrier conditions at the Helmholtz Centre for Infection Research, Braunschweig, Germany, and transferred to the Rodent Centre 1 wk before the infection experiment. Germ-free C57BL/6 mice were bred and infected in the germ-free facility of the Biologisches Zentrallabor. 129Sv/Ev mice used for long-term infection experiments (Figure 4) were transferred to C57BL/6 foster mice at the day of birth, and raised and weaned as usual.

In the streptomycin mouse model, mice were treated with streptomycin (20 mg i.g.) [13] and infected 24 h later with *S.* Tm strains (5 × 10^7^ CFU i.g.) as indicated. For super-infection, L. reuteri RR^Rif^ (8 × 10^6^ CFU i.g.) was administered 24 h after *S.* Tm infection. No streptomycin treatment was performed in spontaneous colitis models and germ-free mice (Figure 3C and 3D).

For induction of acute colitis, CD8^+^ T cells from CL4-TCR transgenic mice that express an α/βT cell receptor recognizing an epitope of the HA protein presented by MHC class I (the H-2Kd:HA512–520 complex) were adoptively transferred into VILLIN-HA mice that express the A/PR8/34 HA epitope from influenza virus A under control of the enterocyte-specific villin promoter [20]. Single-cell suspensions were prepared from the spleen of CL4-TCR transgenic mice. Cell suspensions were depleted of CD4^+^, CD11b^+^, CD45R^+^, DX5^+^, and Ter-119^+^ cells by using the MACS CD8 T cell isolation kit (Miltenyi Biotec, http://www.miltenyibiotec.com/). CL4-TCR T cells were purified by negative selection according to the manufacturer's instructions. Isolated CD8^+^ T cells were washed once in PBS and resuspended (4 × 10^7^ cells/ml of PBS). Then 4 × 10^6^ purified CL4-TCR transgenic T cells were injected intravenously into VILLIN-HA transgenic mice. Disease symptoms (weight loss and diarrhoea) were observed 4–5 d after adoptive transfer.

### Bacteria.

The streptomycin-resistant wild-type strain *S.* Tm^wt^ (SL1344 wild-type [46]) and the isogenic mutant *S.* Tm^avir^ (Δ*invG sseD::aphT; kan* ^R^ [13]) were grown in LB 0.3 M NaCl as described [13]. Colonization was defined by plating on MacConkey agar plates (Oxoid, http://www.oxoid.com/; 100 μg/ml streptomycin). Co-infections with *S.* Tm^avir^ were evaluated by replica-plating on medium containing kanamycin (50 μg/ml).

Culturable intestinal microbiota were grown on Wilkins Chalgren agar supplemented with 5% defibrillated sheep blood (Oxoid) for 3–5 d in an atmosphere of 7% H_2_, 10% CO_2_, and 83% N_2_ at 37 °C in anaerobic jars. 16S rRNA gene sequencing was performed as described below. L. reuteri RR^Rif^ was selected on MRS medium (100 μg/ml of rifampicin; Laboratoire Labo'Life, http://www.labolife.com/) and grown anaerobically.

### Analysis of bacterial loads in intestinal content and systemic organs.

Fresh fecal pellets collected from individual mice and cecum content were resuspended in PBS. Mesenteric lymph nodes (mLN), spleen, and liver were removed aseptically and homogenized in cold PBS (0.5% tergitol and 0.5% BSA). Bacteria were enumerated by plating on appropriate medium.

### Bacterial 16S rRNA gene amplification.

Colonies were isolated and purified twice on Wilkins Chalgren agar (5% sheep blood). DNA was recovered by lysis (Tris/EDTA; 0.5% SDS and 0.1 mg/ml of proteinase K; 37 °C; 1 h), CTAB treatment (1%; 62.5 mM NaCl; 65 °C; 10 min), phenol-chloroform extraction, and 2-propanol precipitation. Broad-range bacterial primers fD_1_ (5′-AGA GTT TGA TCC TGG CTC AG-3′) and rP_1_ (5′-ACG GTT ACC TTG TTA GCA CTT-3′) [47] were used for 16S rRNA gene PCR amplification (94 °C, 5 min; 35 cycles of 94 °C, 1 min; 43 °C, 1 min; 72 °C, 2 min; and 7-min final extension at 72 °C). The PCR product was purified and sequenced with primer rP_1_.

### Quantification of cultured bacteria.

First, bacteria were grouped according to colony morphology. Then, representative colonies were typed by 16S rRNA gene sequencing and comparison to the Ribosomal Database Project II [48]. This allowed a rough estimation of the abundance of the respective bacterial species (Table 1). Two mice were analyzed per condition (*S.* Tm^wt^ and *S.* Tm^avir^ infection day 4 p.i.). Six colony morphological groups were assigned for *S.* Tm^wt^ infection, and ten for *S.* Tm^avir^ infection.

### Histopathological evaluation.

Tissues were cryo-embedded in Tissue Tek OCT Compound (Sysmex, http://www.sysmex-europe.com/), 5-μm cryosections were stained with hematoxylin and eosin (HE), and cecum pathology was evaluated using a histopathological scoring scheme as previously described [49,50] (see Figure S1). Evaluation scored submucosal edema (score 0–3), polymorphonuclear leukocyte infiltration into the lamina propria (score 0–4), loss of goblet cells (score 0–3), and epithelial damage (score 0–3). The combined pathological score for each tissue sample was determined as the sum of these averaged scores: 0–3, no to minimal signs of inflammation that are not sign of a disease (this is frequently found in the cecum of SPF mice); 4–8, moderate inflammation; and 9–13, profound inflammation.

### Immunofluorescence microscopy.

Cecal tissues were fixed in PBS (4% paraformaldehyde [pH 7.4]; 4 °C; 12 h), washed in PBS, equilibrated in PBS (20% sucrose and 0.02% NaN_3_; 4 °C; 12 h) and cryo-embedded in OCT. Cryosections (7 μm) were mounted on glass slides, air-dried (21 °C; 2 h), fixed in PBS (4% paraformaldehyde, 5 min), washed, and blocked with 10% (w/v) normal goat serum in PBS (1 h). *S*. Tm was stained with polyclonal rabbit anti–*Salmonella* O antigen group B serum (factors 1, 4, 5, and 12, Brunschwig, http://www.brunschwig-ch.com/; 1:500 in PBS, 10% goat serum) and a Cy3-conjugated goat anti-rabbit antibody (Milan; 1:300 in PBS, 10% goat serum). The specificity of the anti–*Samonella* O (1, 4 ,5, and 12) antiserum was checked extensively by immunofluorescence microscopy. This was done by analyzing cecum tissue sections from uninfected mice (negative), *S.* Tm–infected mice (positive), S. enterica serovar Enteritidis–infected mice (negative; the LPS of this serovar does not react with this antiserum), and mice with >10 different commensal species, including commensal E. coli strains from our mouse colony, grown in vitro (all negative). DNA was stained with Sytox green (0.1 μg/ml; Sigma-Aldrich, http://www.sigmaaldrich.com/) and F-Actin with Alexa-647-phalloidin (Molecular Probes, http://probes.invitrogen.com/). Sections were mounted with Vectashield hard set (Vector Laboratories, http://www.vectorlabs.com/) and sealed with nail polish. Images were recorded using a PerkinElmer (http://www.perkinelmer.com/) Ultraview confocal imaging system and a Zeiss (http://www.zeiss.com/) Axiovert 200 microscope. For quantification of total bacterial numbers, cecal contents were weighed, fixed in 4% paraformaldehyde, and stained with Sytox green (0.1 μg/ml). Bacteria were counted in a Neubauer's counting chamber using an upright fluorescence microscope (Zeiss).

### Broad-range bacterial 16S rRNA gene sequence analysis.

Total DNA was extracted from cecal contents using a QIAmp DNA stool mini kit (Qiagen, http://www1.qiagen.com/) and a Tissuelyzer device (Qiagen). 16S rRNA genes were amplified by PCR using primers Bact-7F (5′-AGA GTT TGA TYM TGG CTC AG-3′) and Bact-1510R (5′-ACG GYT ACC TTG TTA CGA CTT-3′) and the following cycling conditions: 95 °C, 5 min; 22 cycles of 95 °C, 30 s; 58 °C, 30 s; 72 °C, 2 min; followed by 72 °C, 8 min; 4 °C, ∞. Reaction conditions (100 μl) were as follows: 50 mM KCl, 10 mM Tris-HCl (pH 8.3), 1.5 mM Mg^2+^, 0.2 mM dNTPs, 40 pmol of each primer, and 5 U of Taq DNA polymerase (Eppendorf, http://www.eppendorf.com/). Fragments were purified by gel electrophoresis, excised, recovered using the gene clean kit (Qbiogene; http://www.qbiogene.com/) and dried. The PCR products were suspended in 10 μl of sterile distilled water and between 2 and 5 μl was ligated into pGEM-T Easy Vectors (Promega, http://www.promega.com/). The ligated vectors were transformed into high-efficiency competent JM109 E. coli cells (Promega), plated on LB-carbenicillin agar, and subjected to blue-white screening of colonies. White colonies were picked into 96-well boxes containing 500 μl of Circlegrow medium (Qbiogene, http://www.qbiogene.com/) per well and grown overnight at 37 °C, and the plasmid DNA was then prepped using a modified semi-automated alkaline lysis method. Sequencing was carried out using Applied Biosystems (http://www.appliedbiosystems.com/) BigDye terminators (version 3.1) and run on Applied Biosystems 3730 sequencers. The 16S rRNA gene inserts were sequenced using two primers targeted towards the vector end sequences, M13r (5′-CAGGAAACAGCTATGACC-3′) and T7f (5′-TAATACGACTCACTATAGGG-3′), and one towards an internal region of the gene, 926r (5′-CCGTCAATTC[A/C]TTT[A/G]AGT-3′), in order to bridge any gaps between the sequences generated from the two end primers.

Contigs were built from each three-primer set of sequences using the GAP4 software package [51] and converted to “sense” orientation using OrientationChecker software [52]. These files were then aligned using MUSCLE [53], and the alignments were manually inspected and corrected using the sequence editor function in the ARB package [54]. The files were then tested for the presence of chimeric sequences using Mallard [52] and Bellerophon [55], and putative chimeras were checked using Pintail [56] and BLAST [57]. Positively identified chimeras were removed, and the remaining sequences were examined with the Classifier function at the Ribosomal Database Project II Web site [48] in order to give a broad classification at the phylum level. To obtain more detailed taxonomic information the sequences were divided into phylotypes by generating distance matrices in ARB (with Olsen correction), which were then entered into the DOTUR program [58] set to the furthest neighbour and 99% similarity settings. The resulting phylotypes were then assigned similarities to nearest neighbours using BLAST.

### Statistical analysis of bacterial colonization and intestinal pathology.

Statistical analyses of viable CFU and pathological scores were performed using the exact Mann-Whitney *U* Test and the SPSS version 14.0 software, as described before [8]. Values of *p* < 0.05 were considered statistically significant. Box-plots were created using GraphPad Prism 4 version 4.03 (GraphPad Software, http://www.graphpad.com/).

### Statistical analysis of microbiota composition.

Differences in the phylogenetic compositions of samples were assessed by first assigning the detected 16S rRNA gene sequences to their respective phyla, and then computing the normalized Euclidean distance between the phyla counts. The observed differences were judged for their statistical significance by performing Monte Carlo randomizations: 16S rRNA gene sequences were shuffled between two samples, such that overall sample sizes and total counts for each phylum were maintained. Euclidean distances were then re-computed, and the fraction of distances larger than or equal to the observed distances determined the *p*-values. Bonferroni correction for multiple testing means that *p*-values below 0.005 indicate statistical significance in Figures 2 and 6 and Table 2.

## Supporting Information

Figure S1 Colitis Score Developed for the Streptomycin-Pretreated Mouse Model for *Salmonella* Colitis [8] Mice were pretreated with a single dose of streptomycin (20 mg i.g.) and 24 h later infected with 5 × 10^7^ CFU of *S.* Tm^avir^ (A) or *S.* Tm^wt^ i.g. (B). Mice were sacrificed 1 d p.i.
Left panels of (A) and (B): macroscopic appearance of the cecum from *S.* Tm^avir^– and *S.* Tm^wt^–infected mice, respectively. Note the reduction in size and purulent cecal content in case of *S.* Tm^wt^–induced colitis.
Middle panels: HE-stained cross-section of ceca shown in left panel (scale bar: 1 mm). Note the submucosal edema (se), which is a characteristic of *S.* Tm^wt^–induced colitis. L, cecal lumen.
Right panels: at higher magnification, large numbers of goblet cells (gc) are observed in the cecal mucosa of healthy mice. Colitis leads to reduced numbers of goblet cells due to pronounced epithelial regeneration. Note infiltrating polymorphonuclear leukocytes and desquamated epithelium in the *S.* Tm^wt^–infected cecum (scale bar: 0.05 mm).
Detailed parameters for colitis score are listed in table at bottom of figure.
(272 KB PDF)

Figure S2 FISH Analysis of Microbiota Manipulation by *S.* Tm^wt^ and *S.* Tm^avir^ in the Streptomycin Mouse Model Cecal contents were fixed in PBS (4% paraformaldehyde [pH 7.4]; 4 °C; 12 h), washed in PBS, applied onto polylysine-coated slides, and air-dried. Bacteria were permeabilized (70.000 U/ml of lysozyme; 5 mM EDTA; 100 mM Tris/HCl [pH 7.5]; 37 °C; 10 min), dehydrated with ethanol, and hybridized with HPLC-purified, 5′-labelled 16S rRNA probes (5% formamide, 90 mM NaCl, 20 mM Tris/HCl [pH 7.5]; 46 °C; 2 h): Eub338-cy5 (5′-GCT GCC TCC CGT AGG AGT-3′; detection of all eubacteria [59]), LGC-cy3 or LGC-fluorescein (5′-TCA CGC GGC GTT GCT C-3′; detection of gram-positive bacteria with low G+C content; Firmicutes [60]), and Bac303-cy3 or Bac303-fluorescein (5′-CCA ATG TGG GGG ACC TT-3′; detection of the Bacteroidales group of the Bacteroidetes [61]). Slides were washed at 48 °C (636 mM NaCl, 5 mM EDTA, 0.01% SDS, 20 mM Tris/HCl [pH 7.5]) as described [59]. *S.* Tm was detected by immunostaining (see above), and FISH detection was performed using the Eub338-cy5 probe. The relative abundance of Firmicutes, Bacteroidales, and *S.* Tm was determined by co-staining and imaging at 630× magnification using a PerkinElmer Ultraview confocal imaging system and a Zeiss Axiovert 200 microscope. For each condition, 500–1,750 bacteria were evaluated.
FISH analysis of cecal microbiota from the mice shown in Figure 2. Cecal contents from unmanipulated mice, from mice at days 1 or 5 after streptomycin treatment (20 mg, i.g.), and from streptomycin-treated mice 4 d after infection with *S.* Tm^avir^ and *S.* Tm^wt^ (5 × 10^7^ CFU i.g.; all *n =* 5) were recovered, fixed on cover slips, and hybridized with Eub338 (all bacteria). Firmicutes and Bacteroidales were recognized by hybridization with LGC and BAC303 probes, respectively, and *S.* Tm by an anti–*S.* Tm LPS antiserum (see Materials and Methods). Firmicutes (green), Eub338^+^ Bac303^−^ LGC^+^; Bacteroidales (yellow), Eub338^+^ Bac303^+^ LGC^−^; *Salmonella* (red with white stripes), Eub338^+^ LPS^+^; “unknown” (grey), Eub338^+^ LGC^−^ Bac303^−^ LPS^−^. Abundance of respective groups is expressed as percentage of total Eub338^+^ bacteria.
The results of the FISH analysis confirmed the results obtained via 16S rRNA gene sequencing (Figure 2). Slight differences in the percent composition of the microbiota with respect to Firmicutes, Bacteroidales, and Salmonella spp. obtained via both methods are attributable to species-specific differences in lysis efficiency and 16S rRNA gene copy number.
(124 KB PDF)

Figure S3 Cecal Histopathology in Acute and Chronic Mouse Colitis Models Shown in Figures 4 and 5
Frozen sections of cecal tissues (5 μm) were stained with HE (scale bar: 200 μm). Acute *Salmonella* colitis was observed in C57BL/6 mice infected with *S.* Tm^wt^ (A) but not with *S.* Tm^avir^ (B) 4 d p.i. (compare with Figure 3A). Chronic *Salmonella* colitis was observed in 129Sv/Ev mice infected with *S.* Tm^wt^ (C) but not with *S.* Tm^avir^ (D) 47 d p.i. (compare with Figure 3B). Genetic predisposition (lack of anti-inflammatory cytokine IL10) leads to sporadic occurrence of colitis in C57/BL6^IL10−/−^ mice (E). However, some C57/BL6^IL10−/−^ mice are not affected (F) (compare with Figure 3C). A large number of C3H/HeJBir^IL10−/−^ mice were affected by cecal inflammation (G), but one was not (H) (compare with Figure 3C). L, cecal lumen; se, submucosal edema.
(735 KB PDF)

Figure S4 Colitis Scores for C57/BL6^IL10−/−^ and C3H/HeJBir^IL10−/−^ Mice (A) Frozen sections of cecal tissues (5 μm) were stained with HE (scale bar: 200 μm). Histopathology was scored with respect to submucosal edema (black), polymorphonuclear leukocyte infiltration (grey), loss of goblet cells (dark grey), and epithelial destruction (light grey). The scoring scheme is shown in Figure S1. Scores are plotted as stacked vertical bars. One animal was sacrificed at the end of day 1 p.i. for humane reasons (marked with †).
(B) Confocal fluorescence microscopy image of cecal lumen reveals normal high microbiota densities. Upper left: C3H/HeJBir^IL10−/−^ animal marked with ‡ in (A). The remaining images show animals described in Figure 6B. Upper right: VILLIN-HA control, *S.* Tm^avir^ infected. Lower left: VILLIN-HA+CL4-CD8 (inflammation), non-infected. Lower-right: VILLIN-HA+CL4-CD8 (inflammation), *S.* Tm^avir^ infected. Bacterial DNA is stained by Sytox green (green) and extracellular *S.* Tm by anti-*S.* Tm LPS antiserum (red). Scale bar: 20 or 50 μm as specified.
(1.8 MB PDF)

Figure S5 *S.* Tm^avir^ Efficiently Colonizes Germ-Free Mice Germ-free C57BL/6 mice (*n =* 8) were infected with *S.* Tm^avir^ (5 × 10^7^ CFU i.g.) and sacrificed at day 2 or 4 p.i. (open blue boxes). For comparison, previous data [62] from five mice infected for 1 d with *S.* Tm^wt^ are included (open red boxes). *S.* Tm colonization was analyzed in the cecum content (day 2 p.i.), and cecum pathology was scored (see Material and Methods). Detection limits (dotted line): cecum, 10 CFU/g; mLN, 10 CFU/organ; spleen, 20 CFU/organ. At day 4 p.i., *S.* Tm^avir^ colonization levels in germ-free mice in the absence of re-growing microbiota were significantly higher when compared to streptomycin-treated SPF mice (*p =* 0.002; compare with Figure 3A, left panel).
(105 KB PDF)

Figure S6 16S rRNA Gene Sequence Analysis of Microbiota in VILLIN-HA^CL4-CD8^ Model Visual depiction of the microbiota composition of individual mice. The animals were grouped based on the similarity of their microbiota composition at the phylum level (using the Canberra distance as metric). The resulting groupings are depicted as a dendrogram, and observed phylum counts for each mouse are shown as a heat map (0%–100% of all identified 16S rRNA gene sequences). Labels give unique mouse identifier numbers. The experimental groups are indicated.
(64 KB PDF)

Table S1 Broad-Range Bacterial 16S rRNA Gene Sequence Analysis of the Microbiota Composition from the Experiment Shown in Figures 2 and 6
(277 KB XLS)

Table S2 Phylum-Level Comparison of Microbiota of VILLIN-HA^CL4-CD8^ Model from the Experiment Described in Figure 6
(35 KB DOC)

### Accession Numbers

The GenBank (http://www.ncbi.nlm.nih.gov/Genbank/) accession numbers for the 16S RNA gene sequences shown in Figure 2 are EF604903–EF605247, and for those shown in Figure 6C are EF604904–EF605247 and EU006095–EU006496.

## Abbreviations

CFU: colony-forming units
FISH: fluorescence in situ hybridization
HE: hematoxylin and eosin
IBD: inflammatory bowel disease
i.g.: intragastric
L. reuteri RR^Rif^: rifampicin-resistant variant of Lactobacillus reuteri strain RR
mLN: mesenteric lymph nodes
p.i.: post-infection
*S*. Tm: Salmonella enterica subspecies 1 serovar Typhimurium
*S*. Tm^avir^: Salmonella enterica subspecies 1 serovar Typhimurium Δ*invG sseD::aphT*
*S*. Tm^wt^: wild-type Salmonella enterica subspecies 1 serovar Typhimurium
SPF: specified pathogen free
