# Supplementary material for: Salmonella enterica Serovar Typhimurium Exploits Inflammation to Compete with the Intestinal Microbiota
Source: PLoS Biol. 2007 Aug 28;5(10):e244. doi: 10.1371/journal.pbio.0050244 (PMC1951780; doi:10.1371/journal.pbio.0050244)
Supplement: Supplementary file 1 [file pbio.0050244.sg001.pdf]

Stecher et *al.*, Fig. S1

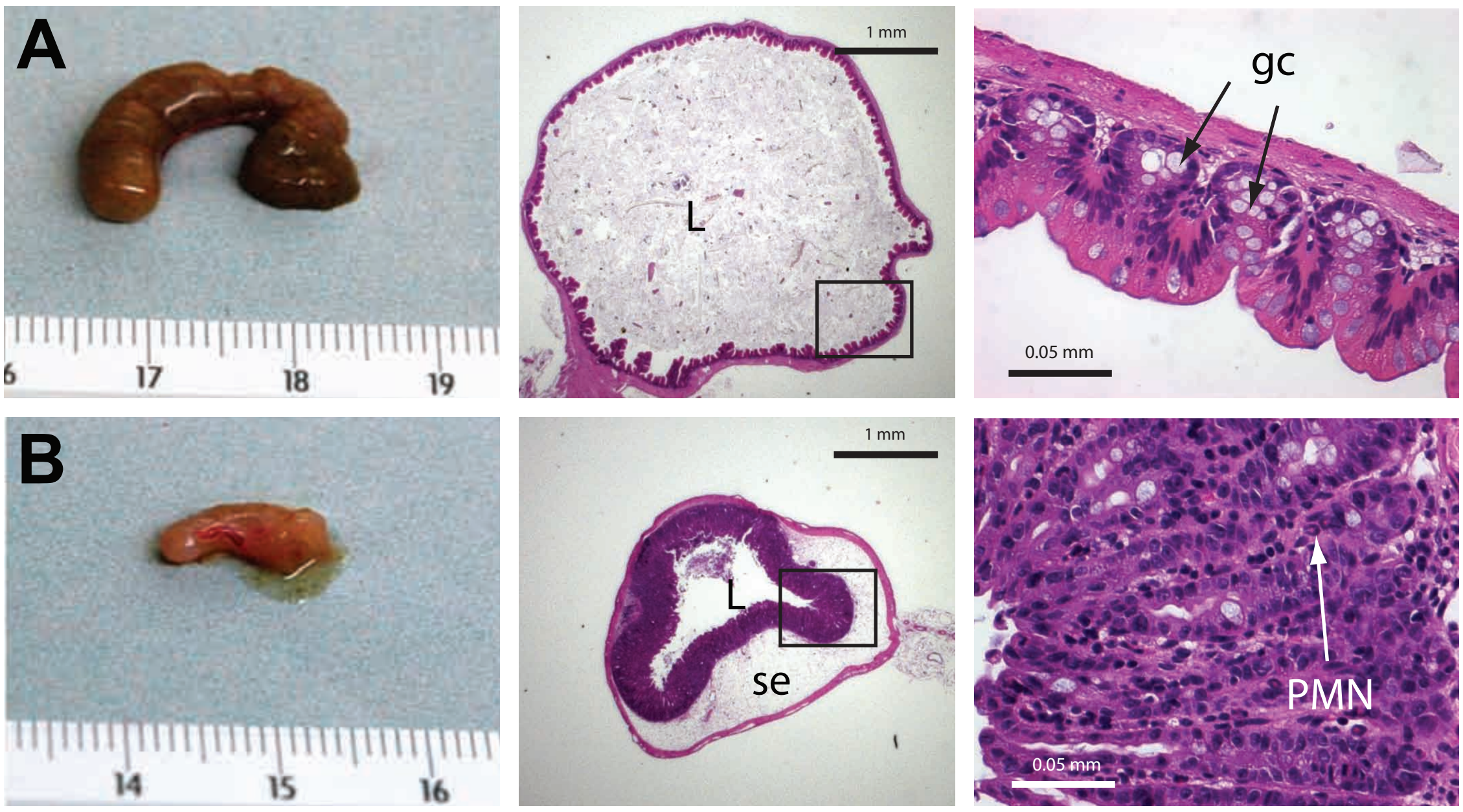

Colitis Score

Parameter	*0*	*1*	*2*	*3*	*4*
submucosal edema (se)	no changes	detectable edema (se<10%)	moderate edema (se=10-40%)	profound edema (se>40%)	
PMN infiltration per 400 x high power field	0-5	6-20	21-60	61-100	> 100
Loss of goblet cells (gc) per 400 x high power field	> 28	11-28	1-10	< 1	
Epithelium	no changes	desquamation	Erosion (gaps of 1-10 cells)	Ulceration (gaps of >10 cells)	
