# Supplementary material for: Salmonella enterica Serovar Typhimurium Exploits Inflammation to Compete with the Intestinal Microbiota
Source: PLoS Biol. 2007 Aug 28;5(10):e244. doi: 10.1371/journal.pbio.0050244 (PMC1951780; doi:10.1371/journal.pbio.0050244)

Stecher et *al.*, Fig. S3

S. Tm-induced colitis

S. Tm^{wt}
C57BL/6
day 4 p.i.

S. Tm^{avir}
C57BL/6
day 4 p.i.

S. Tm^{wt}
129Sv/Ev
day 47 p.i.

S. Tm^{avir}
129Sv/Ev
day 47 p.i.

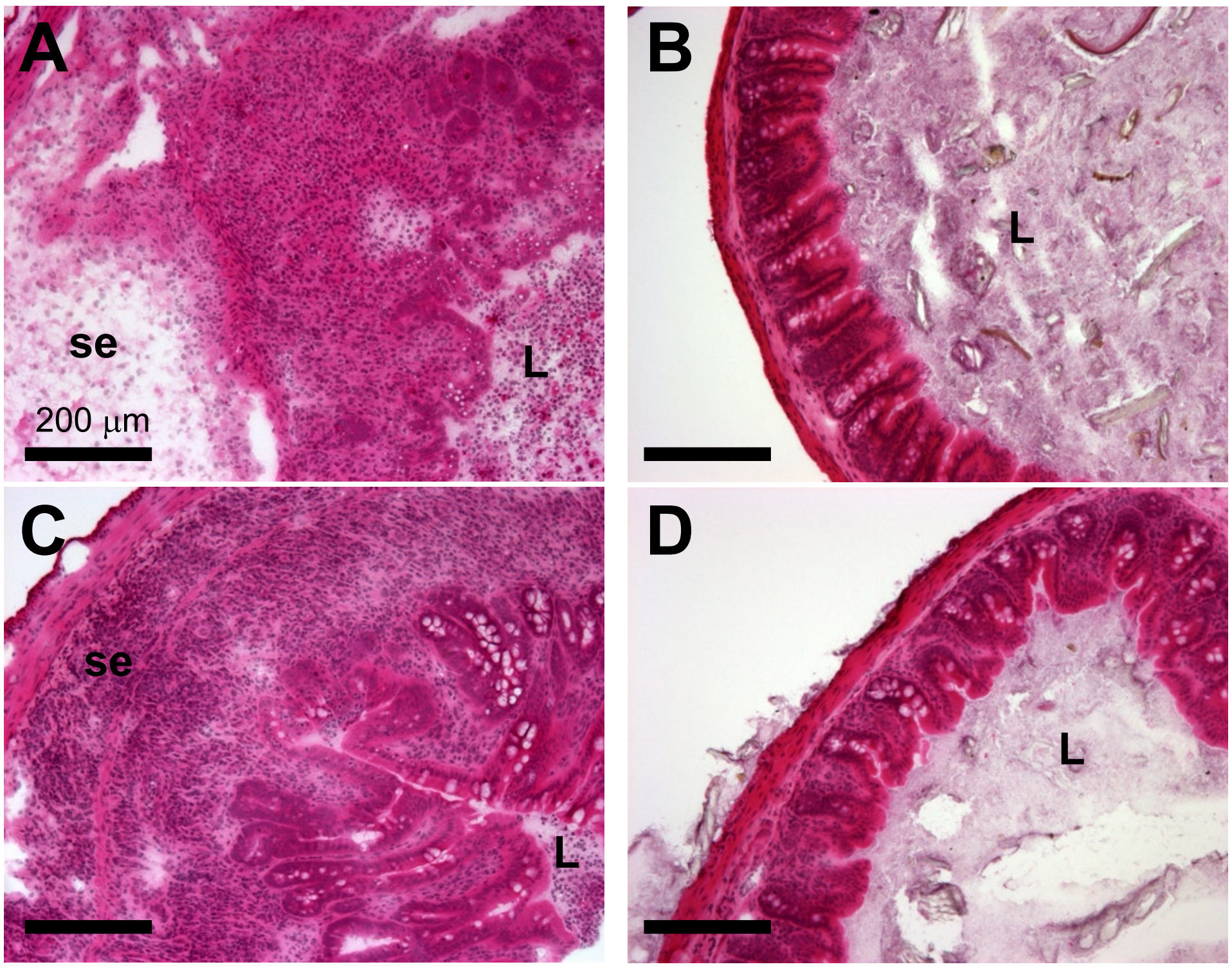

Colitis in IL10$^{-/-}$ mouse models

S. Tm^{avir}
C57BL/6$^{IL10-/-}$
day 2 p.i.

S. Tm^{avir}
C57BL/6$^{IL10-/-}$
day 2 p.i.

S. Tm^{avir}
C3H/HeBir$^{IL10-/-}$
day 2 p.i.

S. Tm^{avir}
C3H/HeBir$^{IL10-/-}$
day 2 p.i.

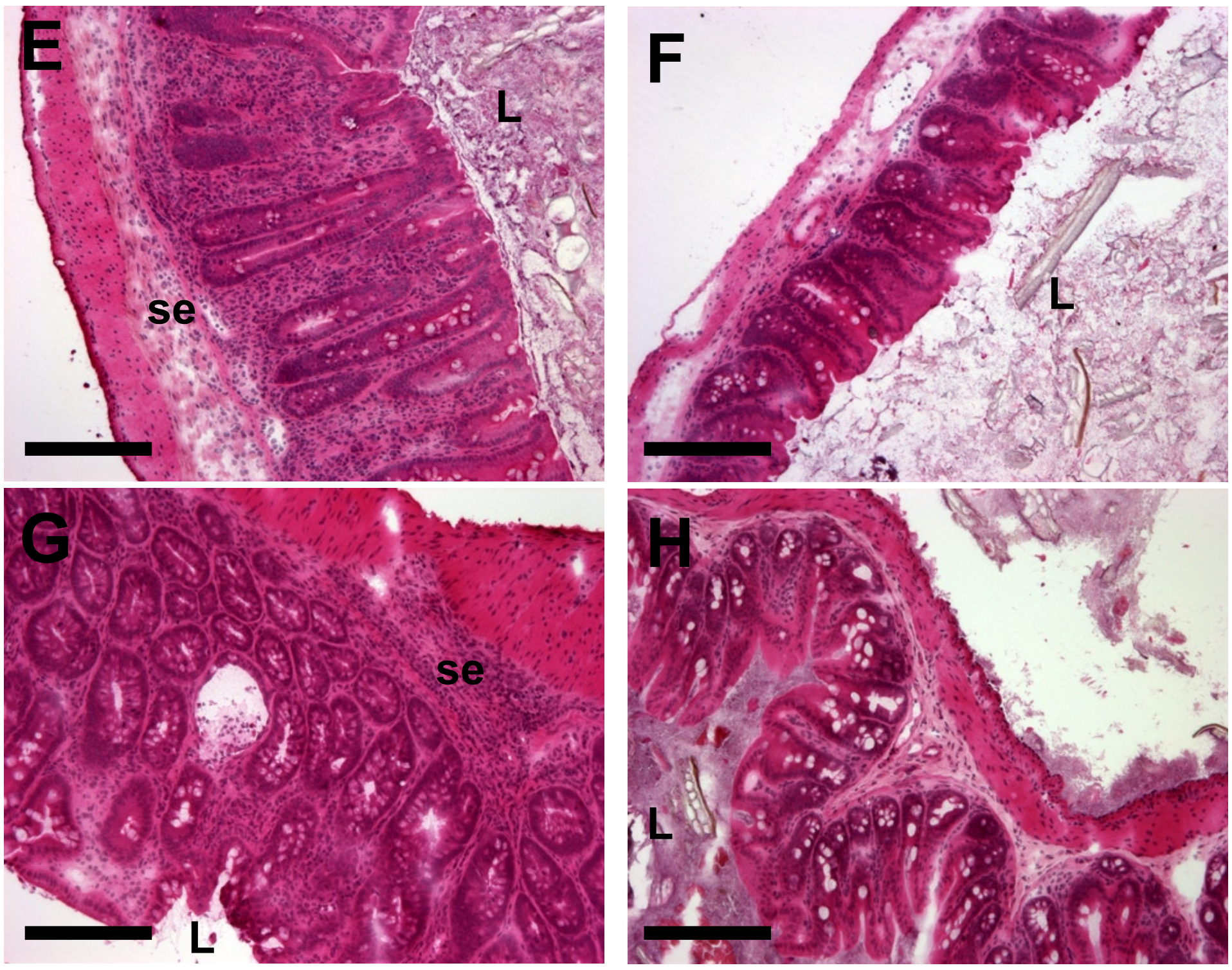

Supplement: Supplementary file 3 [file pbio.0050244.sg003.pdf]
