# Supplementary material for: Salmonella enterica Serovar Typhimurium Exploits Inflammation to Compete with the Intestinal Microbiota
Source: PLoS Biol. 2007 Aug 28;5(10):e244. doi: 10.1371/journal.pbio.0050244 (PMC1951780; doi:10.1371/journal.pbio.0050244)

Stecher et *al.*, Fig. S5

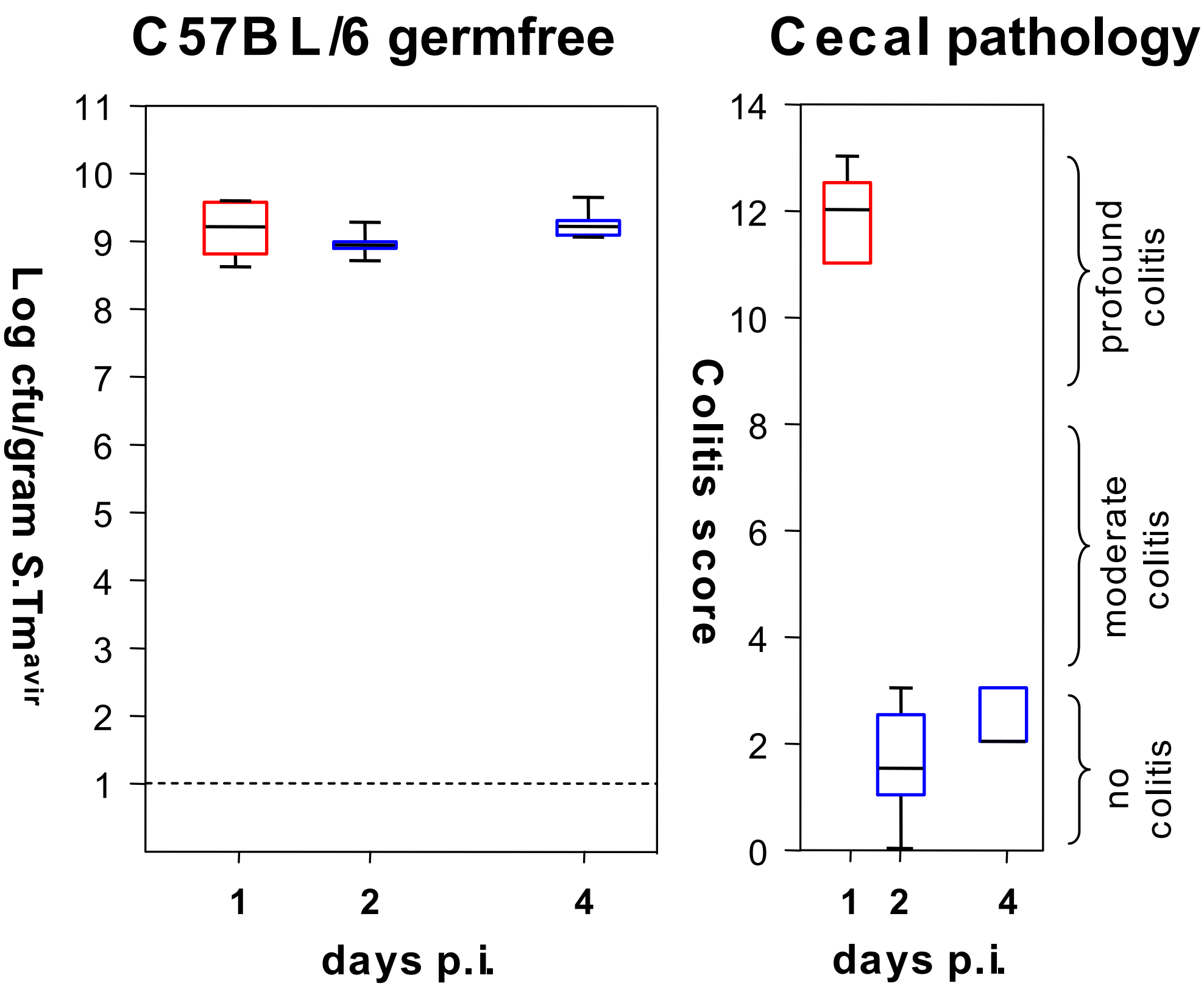

Supplement: Supplementary file 5 [file pbio.0050244.sg005.pdf]
