# Supplementary material for: Salmonella enterica Serovar Typhimurium Exploits Inflammation to Compete with the Intestinal Microbiota
Source: PLoS Biol. 2007 Aug 28;5(10):e244. doi: 10.1371/journal.pbio.0050244 (PMC1951780; doi:10.1371/journal.pbio.0050244)
Supplement: Supplementary file 7 [file pbio.0050244.st002.doc]

**Table S2.** Phylum-level comparison of microbiota VILLIN-HA^CL4-CD8^ model from the experiment described in Fig. 6

| **Group #1** | **Group #2** | **p-value** | *Difference* |
| --- | --- | --- | --- |
| *S.* Tm, no colitis | no *S.* Tm, no colitis | 0.32664 | << indistinguishable |
| no *S.* Tm, colitis | *S.* Tm, no colitis | 0.00562 | << borderline distinguishable |
| no *S.* Tm, colitis | no *S.* Tm, no colitis | 0.00155 | << borderline distinguishable |
| no *S.* Tm*,* colitis | *S.* Tm, colitis | < 0.00001 | << clear differences |
| *S.* Tm, colitis | *S.* Tm, no colitis | < 0.00001 | << clear differences |
| *S.* Tm, colitis | no *S.* Tm, no colitis | < 0.00001 | << clear differences |
|  |  |  |  |

p≥ 0.005: no significant difference detectable (materials and methods).
